# Restoration of mutant bestrophin-1 expression, localisation and function in a polarised epithelial cell model

**DOI:** 10.1242/dmm.024216

**Published:** 2016-11-01

**Authors:** Carolina Uggenti, Kit Briant, Anne-Kathrin Streit, Steven Thomson, Yee Hui Koay, Richard A. Baines, Eileithyia Swanton, Forbes D. Manson

**Affiliations:** Faculty of Biology, Medicine and Health, The University of Manchester, Manchester M13 9PT, UK

**Keywords:** Autosomal recessive bestrophinopathy, Bestrophin-1, Chemical chaperone, 4-phenylbutyrate

## Abstract

Autosomal recessive bestrophinopathy (ARB) is a retinopathy caused by mutations in the bestrophin-1 protein, which is thought to function as a Ca^2+^-gated Cl^−^ channel in the basolateral surface of the retinal pigment epithelium (RPE). Using a stably transfected polarised epithelial cell model, we show that four ARB mutant bestrophin-1 proteins were mislocalised and subjected to proteasomal degradation. In contrast to the wild-type bestrophin-1, each of the four mutant proteins also failed to conduct Cl^−^ ions in transiently transfected cells as determined by whole-cell patch clamp. We demonstrate that a combination of two clinically approved drugs, bortezomib and 4-phenylbutyrate (4PBA), successfully restored the expression and localisation of all four ARB mutant bestrophin-1 proteins. Importantly, the Cl^−^ conductance function of each of the mutant bestrophin-1 proteins was fully restored to that of wild-type bestrophin-1 by treatment of cells with 4PBA alone. The functional rescue achieved with 4PBA is significant because it suggests that this drug, which is already approved for long-term use in infants and adults, might represent a promising therapy for the treatment of ARB and other bestrophinopathies resulting from missense mutations in *BEST1*.

## Abstract (precis)

**Summary:** Chemical chaperone 4PBA fully restores Cl^−^ conductance activity for mutant bestrophin-1 proteins associated with inherited retinal dystrophy, autosomal recessive bestrophinopathy.

## INTRODUCTION

Bestrophin-1 is a homopentameric channel protein primarily expressed in the basolateral membrane of the retinal pigment epithelium (RPE) (Marmorstein et al., 2000; Kane Dickson et al., 2014). Over 250 variants in the bestrophin-1 gene, *BEST1*, are associated with a group of retinal degenerative diseases collectively known as bestrophinopathies [Best disease, autosomal dominant vitreoretinochoroidopathy, retinitis pigmentosa (RP), autosomal recessive bestrophinopathy (ARB)] (Burgess et al., 2008). All the bestrophinopathies are associated with abnormal RPE function as measured by an electro-oculogram, but the effect on retinal function and the age of onset is variable (Boon et al., 2009).

The endogenous activity of bestrophin-1 remains unclear. Exogenous expression of bestrophin-1 results in a novel Ca^2+^-activated Cl^−^ current (Sun et al., 2002); it is also highly permeable to HCO_3_ (Qu and Hartzell, 2008) and has been reported to act as a glutamate channel in hippocampal astrocytes (Woo et al., 2012). These data, together with the crystal structure of bestrophin-1 orthologues, suggest that it is an anion channel (Kane Dickson et al., 2014). A common consequence of mutations in bestrophin-1 is aberrant localisation and reduced stability of the mutant proteins (Davidson et al., 2011; Johnson et al., 2014). ARB is an autosomal recessive disease and the associated missense mutations have a loss-of-function effect on bestrophin-1 activity. In previous studies into bestrophin-1 mutations associated with ARB we found that all of the nine mutant proteins we tested had a significantly smaller Cl^−^ conductance compared with wild-type (WT) bestrophin-1 (Burgess et al., 2008; Davidson et al., 2009, 2011). In addition, most mutant proteins were mislocalised (7 of 9 examined), and had reduced stability resulting from proteasomal degradation (6 of 9 examined) (Burgess et al., 2008; Davidson et al., 2009, 2011). Thus, these ARB-associated mutations seem to disrupt bestrophin-1 function, at least in part, by disrupting the protein's folding, leading to retention and degradation by the quality control systems that eliminate misfolded proteins from the secretory pathway (Christianson and Ye, 2014).

Some mutant proteins, including ion channels, retain functional activity and can be rescued from their misfolded state by chemical compounds that enhance the capacity of cellular protein folding pathways (proteostasis regulators) or stabilise protein folds (chemical and pharmacological chaperones). Such approaches have shown success in the laboratory for a variety of genetic diseases including α1-anti-trypsin deficiency, cystic fibrosis, and Huntington's disease, using compounds such as 4-phenylbutyrate (4PBA), carbamazepine and celastrol (Lim et al., 2004; Singh et al., 2008; Puls et al., 2013; Cleren et al., 2005). In the context of eye diseases, treatment with the chemical chaperone tauroursodeoxycholate (TUDCA) preserved retina function and structure in rodent models of Bardet–Biedl syndrome, RP and Leber congenital amaurosis (Drack et al., 2012; Phillips et al., 2008; Zhang et al., 2012; Fernández-Sánchez et al., 2011), whilst the application of topical 4PBA restored normal intraocular pressure in a transgenic myocilin mouse model of primary open angle glaucoma (Zode et al., 2012). These recent advances suggest that manipulation of the cell's protein folding pathways might also present an opportunity to restore mutant bestrophin-1 localization and/or function.

Here, we report the use of chemical chaperones and proteostasis regulators to enhance the expression of ARB mutant bestrophin-1 proteins, correct their trafficking to the plasma membrane, and restore Cl^−^ conductance functionality.

## RESULTS

### ARB-associated mutations disrupt bestrophin-1 expression and localisation in polarised epithelial cells

**Fig. 1.**
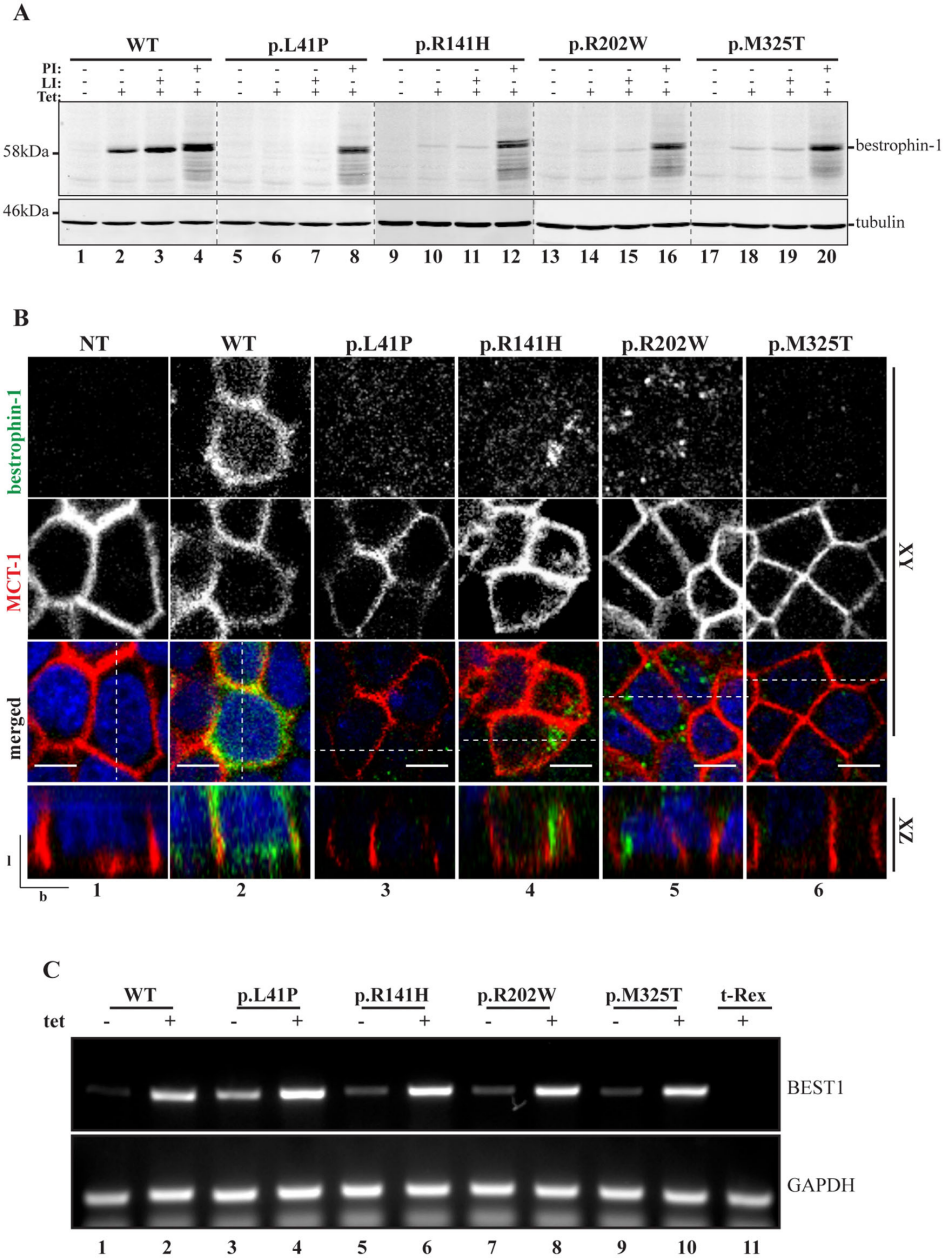
**Expression of WT and ARB-associated bestrophin-1 in stable MDCKII cells.** (A) MDCKII cells were grown on Transwell filters until they formed a confluent, polarised monolayer, then WT or mutant p.L41P, p.R141H, p.R202W and p.M325T bestrophin-1 expression was induced with tetracycline (Tet) for 24 h before direct lysis in SDS sample buffer for western blotting analysis. Cells were tested for the expression of bestrophin-1 in presence or absence of tetracycline, lysosomal protease inhibitors (LI) and proteasome inhibitors (PI). An anti-tubulin antibody was used as a loading control. The expression of WT bestrophin-1 was significantly increased by treating cells with PI following Tet induction (untreated, *n*=3; PI-treated, *n*=3; *P*=0.0189 by one-way ANOVA, followed by Bonferroni multi-comparison test). PI treatment led to a significant increase in the amount of each of the mutant bestrophin-1 proteins (p.L41P with and without PI, *n*=3, *P*=0.0012; p.R141H with and without PI, *n*=3, *P*=0.0042; p.R202W with and without PI, *n*=3, *P*=0.0107; p.M325T with and without PI, *n*=3, *P*=0.0037; analysed by one-way ANOVA, followed by Bonferroni multi-comparison test). (B) Localisation of WT or ARB-causing bestrophin-1 was determined by confocal immunofluorescence microscopy. Cells were fixed with 4% PFA, permeabilised with 0.1% Triton X-100 and stained for bestrophin-1 (green) and monocarboxylate transporter 1 (MCT1, red), which was used as a marker for the basolateral plasma membrane. Representative *XY* and *XZ* scans are shown. Scale bar: 5 µm. l, lateral; b, basal. Dotted line in merged images shows position of the *XZ* scan. (C) Relative WT and mutant *BEST1* mRNA levels were determined for stable MDCKII lines before and after tetracycline induction by semi-quantitative RT-PCR. Data are expressed relative to GAPDH and are representative of three independent replicates.

In order to provide a cellular model with which to examine the ability of selected compounds to rescue mutant bestrophin-1 expression and localisation, we generated tetracycline-inducible MDCKII cell lines stably expressing WT bestrophin-1 and four ARB-associated mutants (p.L41P, p.R141H, p.R202W and p.M325T). MDCK II cells adopt a polarised morphology typical of epithelial cells that we and others have shown can recapitulate the authentic localisation of WT bestrophin-1 (Mostov et al., 2003; Davidson et al., 2009; Milenkovic et al., 2011; Doumanov et al., 2013; Johnson et al., 2013). MDCKII stable cells lines were grown on Transwell porous filters for 5-6 days until they formed a confluent monolayer, then bestrophin-1 expression was induced by addition of tetracycline. After 24 h of induction, bestrophin-1 protein could be detected in cell lysates as a single band at the expected molecular mass of 68 kDa by immunoblotting with a monoclonal anti-bestrophin-1 antibody (Fig. 1A, lane 2). Bestrophin-1 was not observed in the absence of tetracycline (Fig. 1A, lane 1), confirming that transgene expression was tightly regulated and that MDCKII cells do not express detectable levels of endogenous bestrophin-1. Indirect confocal immunofluorescence microscopy confirmed that the MDCKII cells were polarised, and that WT bestrophin-1 was localised to the basolateral surfaces of the plasma membrane where it co-localised with MCT-1 (Fig. 1B, panel 2). In addition, some WT bestrophin-1 staining was observed intracellularly (Fig. 1B, panel 2), suggesting that a proportion of the protein did not reach the cell surface. This might reflect the native distribution of bestrophin-1 (Barro-Soria et al., 2010), or could indicate that a fraction of nascent WT bestrophin-1 does not fold correctly in MDCKII cells and is thus retained by intracellular quality control systems. Consistent with the latter interpretation, the expression level of WT bestrophin-1 was significantly increased by treating cells with the proteasome inhibitor PSII [(benzyloxycarbonyl)-Leu-Leu-phenylalaninal] following tetracycline induction (Fig. 1A, lane 4; Fig. S1). In contrast, inhibition of lysosomal proteases using a combination of leupeptin and pepstatin A caused only a small, non-significant, increase in WT bestrophin-1 levels (Fig. 1A, lane 3; Fig. S1). Together, these results confirm previous findings that MDCKII cells are able to support proper folding and basolateral transport of WT bestrophin-1, but indicate that a proportion of the WT protein is targeted for proteasomal degradation.

In comparison with the WT protein, extremely low levels of p.L41P, p.R141H, p.R202W and p.M325T bestrophin-1 were detected in lysates of MDCKII cell lines stably transfected with these mutant constructs, even after 24 h of tetracycline induction (Fig. 1A, lanes 6, 10, 14, 18). Immunofluorescence microscopy revealed only faint bestrophin-1 staining in these stable cell lines (Fig. 1B, panels 3-6), consistent with low expression levels of the mutant proteins. Furthermore, none of the bestrophin-1 mutants co-localised with MCT-1 at the basolateral membrane, but were instead observed in distinct intracellular structures (Fig. 1B, panels 3-6). Comparable levels of WT and mutant bestrophin-1 mRNA were detected following induction with tetracycline (Fig. 1C), suggesting that the observed differences in protein expression did not reflect altered transcription or mRNA stability. In order to determine whether the reduced steady-state levels of the mutant proteins were instead the result of proteolytic degradation of mislocalised bestrophin-1, cells were induced with tetracycline then treated with inhibitors of proteasomal and lysosomal degradation pathways for 6 h. Inhibition of lysosomal proteases did not significantly affect levels of any of the four mutant bestrophin-1 proteins (Fig. 1A, lanes 7, 11, 15 and 19; Fig. S1). However, treatment with the proteasome inhibitor PSII led to a dramatic and significant increase in the amount of each of the mutant bestrophin-1 proteins that could be detected by immunoblotting (Fig. 1A, lanes 8, 12, 16 and 20; Fig. S1). Together, these results suggest that the four ARB-associated mutations disrupt the folding of bestrophin-1, leading to retention by intracellular quality control systems and degradation via a proteasome-dependent pathway such as ER-associated degradation (ERAD). Similar mechanisms have been shown to limit the expression of a wide range of mutant membrane proteins (Sano and Reed, 2013; Ng et al., 2012), and suggest a model whereby a lack of functional bestrophin-1 protein underlies ARB.

We noted that the accumulated mutant bestrophin-1 following treatment with PSII appeared as a doublet upon SDS-PAGE and immunoblotting, with the additional band being of a slightly greater apparent mass than that detected in untreated cells (Fig. 1A, compare second and fourth lane of each mutant). Bestrophin-1 does not contain any consensus sites for N-glycosylation within its luminal/extracellular domains, and we tested whether this higher molecular weight band was the result of post-translational modification by phosphorylation or mannosylation, but were unable to demonstrate that either modification was present (data not shown). Hence, the nature of this additional form is presently unknown, but as it was also observed upon proteasome inhibitor treatment of cells expressing WT bestrophin-1 (Fig. 1A, compare lanes 2 and 4), it seems likely that it represents a feature of the WT protein.

**Fig. 2.**
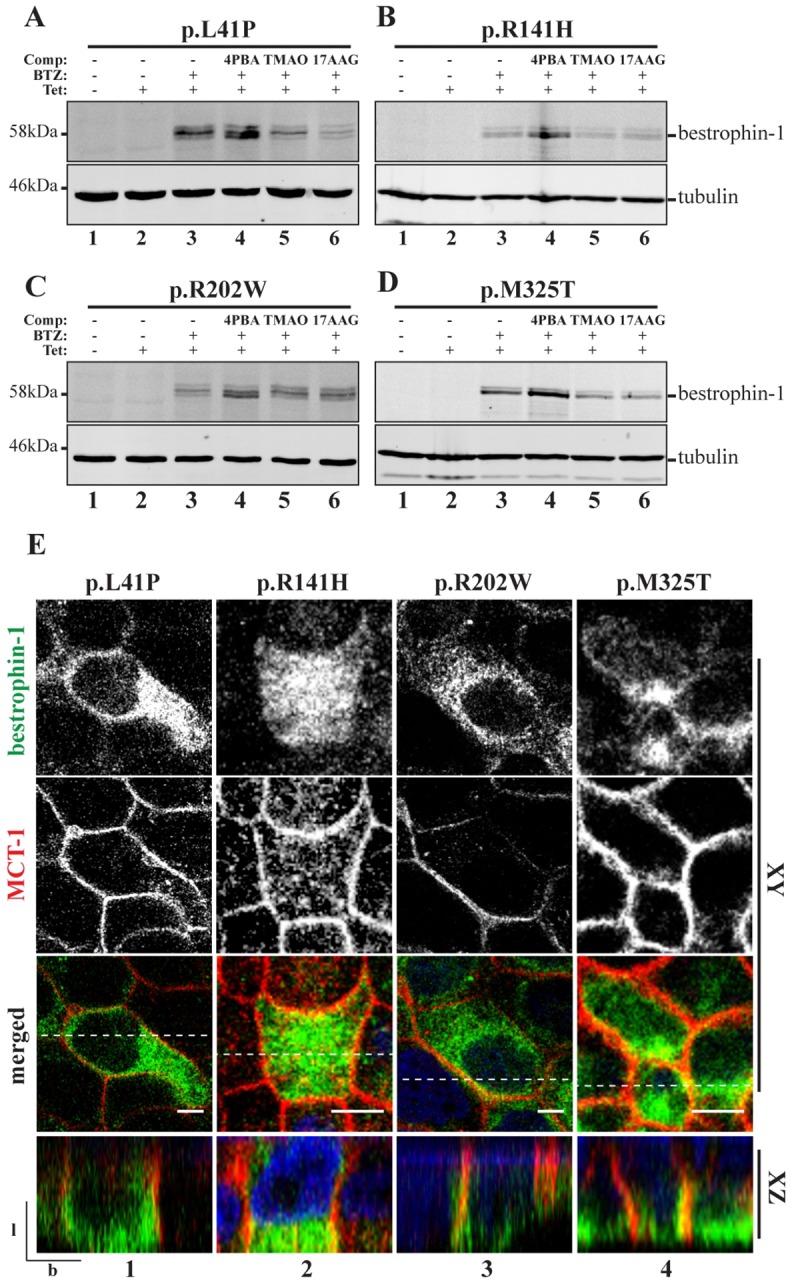
**Small molecule treatment of ARB-associated bestrophin-1 in stable polarised MDCKII cells.** (A-D) Mutant bestrophin-1 proteins expression level was investigated by western blot. p.L41P, p.R141H, p.R202W and p.M325T bestrophin-1 expression was induced with tetracycline (Tet) and cells were treated with BTZ or a combination of BTZ+4PBA, TMAO or 17-AAG for 24 h before direct lysis in SDS sample buffer. An anti-tubulin antibody was used as a loading control. (E) Confocal immunofluorescence analysis was used to investigate mutant bestrophin-1 localisation (green) in MDCKII stable cells lines following 24 h treatment with BTZ+4PBA. Representative *XY* and *YZ* scans for each mutant are shown. Co-localisation with MCT-1 (red) was used as a marker for correct trafficking to the basolateral plasma membrane. Scale bar: 5 µm. l, lateral; b, basal. Dotted line in merged images shows position of the *XZ* scan.

The proteasome inhibitor bortezomib (BTZ, also known as Velcade) is currently in use for the treatment of multiple myeloma (Field-Smith et al., 2006), and we tested whether this inhibitor was also able to restore expression levels of mutant bestrophin-1. Indeed, treatment of cells with 25 nM BTZ substantially increased the levels of all four ARB-associated bestrophin-1 mutant proteins (Fig. 2A-D, lane 3), providing further evidence that they are targeted for ERAD. However, for all four mutant bestrophin-1 proteins the majority of the protein remained intracellular. Inhibiting degradation can in some cases be sufficient to promote proper folding and trafficking of mutant proteins (Wang and Segatori, 2013). This seems to be partly true for the mutant proteins p.L41P and p.R141H, which showed some co-localisation with MCT-1 following BTZ treatment, although most of the mutant protein still seemed to be intracellular (Fig. S2). Similarly, a proportion of p.L41P bestrophin-1 localises to the basolateral membrane of transiently transfected MDCKII cells in the absence of proteasome inhibitors (Davidson et al., 2011; Johnson et al., 2014). These results indicate that degradation of p.L41P and p.R141H bestrophin-1 competes with productive folding, and that reducing degradation or increasing expression level (e.g. using transient transfection, which typically results in higher expression) allows a small pool of the mutant protein to fold and traffic to the cell surface. Although exposure to BTZ also increased the expression levels of the other two mutant proteins, p.R202W and p.M325T, they remained predominantly intracellular and did not co-localise extensively with MCT-1 (Fig. S2). These data support the conclusion that mutant bestrophin-1 is degraded by ERAD and suggest that increasing the cellular concentration of mutant bestrohin-1 is not sufficient to effectively rescue folding and restore proper localisation.

### Restoration of mutant bestrophin-1 expression and localisation

We next examined whether simultaneously inhibiting proteasomal degradation and enhancing folding using chemical chaperones or proteostasis regulators would allow rescue of mutant bestrophin-1 localisation (Mu et al., 2008; Wang et al., 2011). Cells were induced to express bestrophin-1 and treated with a combination of BTZ plus a chemical chaperone or proteostasis regulator for 24 h. At a qualitative level, each of the four bestrophin-1 mutant proteins examined was affected in a similar way by these combinatorial treatments (Fig. 2). Notably, the chemical chaperone 4PBA (2.5 mM) in combination with BTZ caused a further increase in the cellular levels of each mutant protein, above that seen with BTZ alone (Fig. 2A-D, compare lanes 3 and 4). A similar effect was also seen with WT bestrophin-1 (data not shown). The detection of intracellular WT protein after induction (Fig. 1B, panel 2), and its increased expression after treatment with the chemical chaperone 4PBA (Fig. S3), suggest that a proportion of the WT protein is misfolded. In contrast, the other chemical chaperones trimethylamine N-oxide (TMAO, 50 mM; Fig. 2A-D, lane 5) and TUDCA (1 mM; data not shown) and the proteostasis regulators 17-AAG (17-N-allylamino-17-demethoxygeldanamycin) (50 nM; Fig. 2A-D, lane 6) and celastrol (50 nM; data not shown) did not appreciably alter expression levels of mutant bestrophin-1, either alone or in combination with BTZ. 4PBA is a low molecular weight chemical chaperone that non-specifically stabilises native protein folds, and thus enhances the productive folding and trafficking of a number of folding-defective mutant proteins (Powers et al., 2009). The observation that the treatment of cells with 4PBA increased the expression levels of mutant bestrophin-1 indicates that it promotes folding of these disease-associated mutant proteins, thereby reducing their degradation by ERAD.

Because correctly folded bestrophin-1 is transported to the basolateral surface, we examined the subcellular localisation of bestrophin-1 in cells treated with different small molecules. Of the treatments tested we observed that the combination of BTZ plus 4PBA was able to partially restore cell surface localisation to each of the bestrophin-1 mutants (Fig. 2E). Although much of the mutant bestrophin-1 remained intracellular, an increased amount of the mutant protein was also observed at the plasma membrane (Fig. 2E, bottom two rows). This is most evident in the XZ projections, where bestrophin-1 is clearly seen to co-localise with MCT-1 on the basolateral surface (Fig. 2E, bottom row). We conclude that in the presence of BTZ, treatment with 4PBA is able to promote the correct folding and trafficking of mutant bestrophin-1, so restoring its cell surface localisation.

### Restoration of mutant bestrophin-1 function

**Fig. 3.**
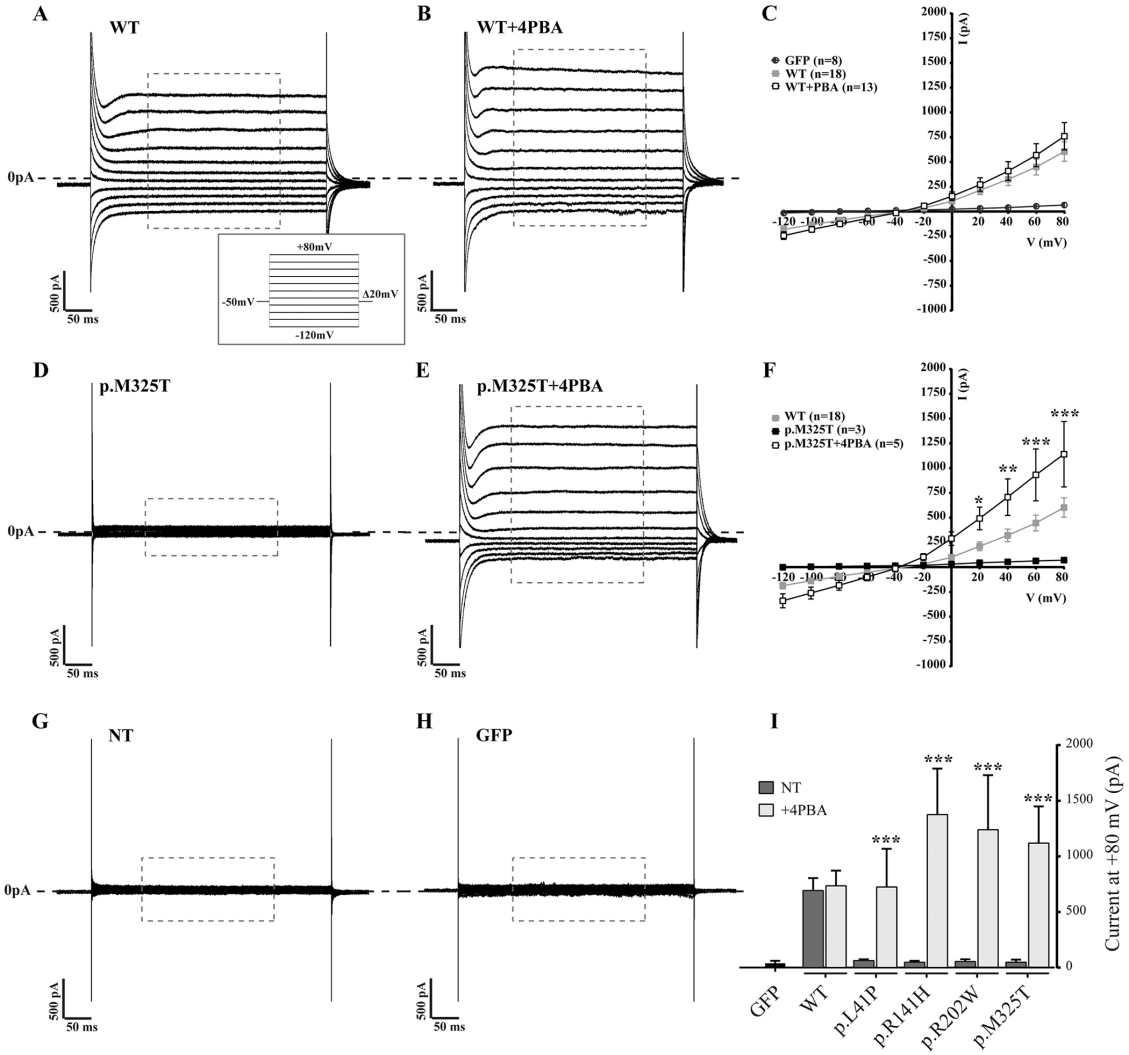
**Whole-cell patch clamp of transiently transfected HEK293 expressing WT and mutant bestrophin-1 after treatment with 4PBA.** Representative whole-cell current responses of HEK293 cells transiently transfected with WT (A,B) or p.M325T (D,E) bestrophin-1 treated with 2.5 mM 4PBA. Recordings were taken from −120 mV to +80 mV in Δ20 mV steps of 450 ms each. The conductance in cells expressing p.M325T was increased from 48.9±23.1 mA (mean±s.e.m., *n*=3) in the absence of 4PBA to 1118.7±330.8 pA (*n*=5) following 4PBA treatment (*P*<0.0001 by two-way ANOVA, followed Turkey multi-comparison test). The Cl^−^ conductance induced by p.M325T in the presence of 4PBA was not significantly different to that that induced by WT bestrophin-1 (580.6±98.7 pA). The holding potential was −50 mV. The mean current/voltage relationship (I/V) is shown for cells transfected with WT with and without 4PBA treatment, compared with transfection with GFP only (C). (I) Plot of Cl^−^ conductance (pA) at a holding potential of +80 mV for WT and 4 ARB-causing mutants before and after treatment with 2.5 mM 4PBA. Results are presented as mean±s.e.m. *P<0.05, **P<0.01 and ***P<0.001 indicate significant differences between treated and untreated cells by one-way ANOVA, followed by Bonferroni multi-comparison test. *n*, number of cells recorded in independent experiments; NT, not treated (–4PBA).

Having established that mutant bestrophin-1 could be rescued from its misfolded state to allow trafficking to the cell surface, we next examined whether the mutated proteins were functional. The best-characterised activity of bestrophin-1 is as an anion channel (Sun et al., 2002; Davidson et al., 2011, 2009). Expression of WT bestrophin-1 induces an exogenous Cl^−^ conductance in HEK293 cells that can be measured by whole-cell patch clamp. We therefore transiently transfected HEK293 cells with the WT and four ARB mutant bestrophin-1 constructs and measured the Cl^−^ conductance in the absence and presence of small molecules. Fig. 3C shows the mean current-voltage relationship obtained from cells transfected with WT bestrophin-1 or, in control cells, transfected only with GFP (a marker for successful transfection). WT bestrophin-1 produced a large time-independent Cl^−^ current with a slightly outwardly rectifying I-V relationship (Fig. 3A). Non-transfected cells produced a very small Cl^−^ current, comparable with that produced by cells transfected with GFP alone (Fig. 3G,H), and in both cases the Cl^−^ current was significantly less than obtained following transfection with WT bestrophin-1 (Fig. 3A,G,H). Cl^−^ currents in cells transiently expressing the four ARB bestrophin-1 mutants were notably reduced compared with those induced by the expression of WT bestrophin-1 at all positive holding potentials (Fig. 3D,F,I; Fig. S4). Interestingly, however, the reversal potentials for the small currents induced by the mutant proteins were comparable with that of the WT bestrophin-1-induced current of −17.6±0.87 mV (mean±s.e.m.) (Fig. 3F; Fig. S4). This observation is consistent with the reduction in Cl^−^ conductance by the ARB-associated mutant proteins resulting from fewer active channels in the cell membrane rather than a change to voltage sensitivity or channel kinetics of the mutant proteins.

**Fig. 4.**
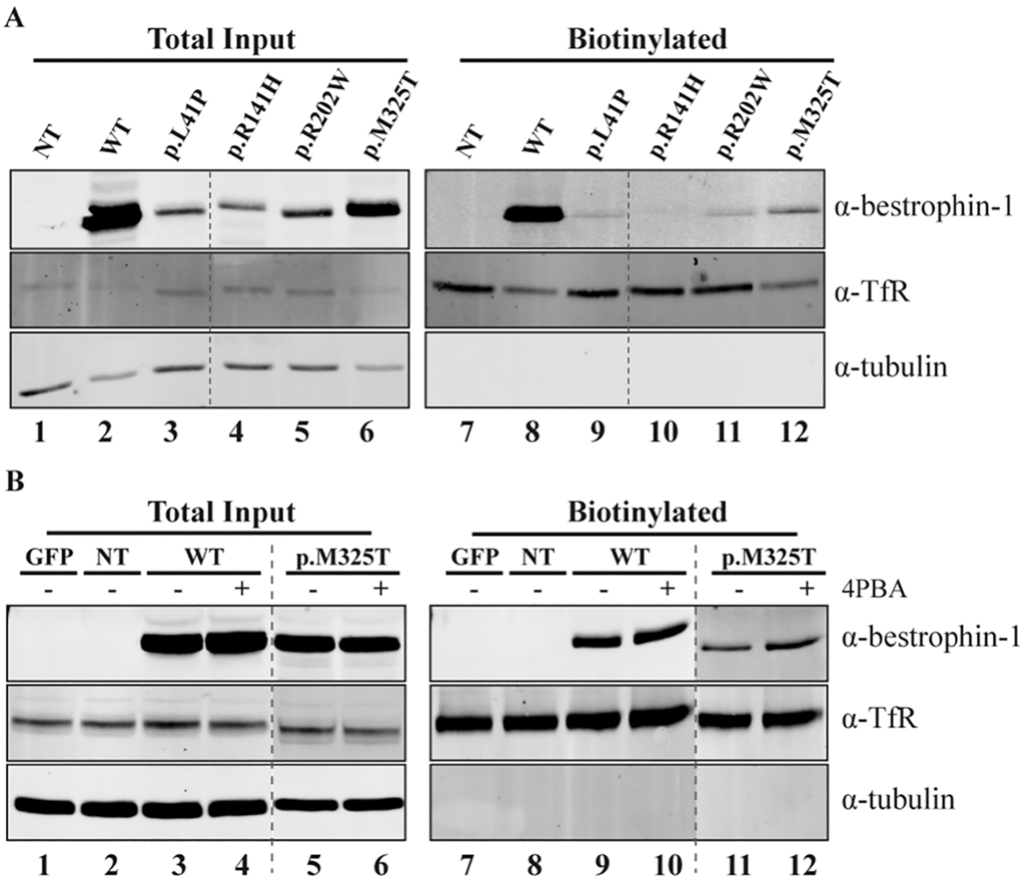
**Biotinylation of WT and mutant bestrophin-1 before and after treatment with 4PBA in transiently transfected HEK293 cells.** (A) Western blot analysis of whole cell lysates (lanes 1-6) and precipitated biotinylated cell surface proteins (lanes 7-12) of cells expressing WT or mutant bestrophin-1 proteins. Cells were labelled with biotin for 30 min. The unreacted biotin was then quenched and the cells lysed. One tenth of the lysate was used for the total input. Biotinylated (cell surface) proteins were precipitated using streptavidin beads. (B) Western blot analysis of whole cell lysates (lanes 1-6) and precipitated biotinylated cell surface proteins (lanes 7-12) of cells expressing WT or p.M325T bestrophin-1. Cells were grown in absence (lanes 1-3, 5,7-9,11) or presence (lanes 4,6,10,12) of 4PBA for 24 h following transfection, before labelling with biotin. An anti-transferrin receptor (α-TfR) antibody was used as a control for the biotinylation assay. An anti-tubulin antibody was used as loading control. GFP, cells transfected with GFP only; NT, non transfected cells.

As reported previously (Milenkovic et al., 2009; Davidson et al., 2009), the large majority of WT bestrophin-1 transiently expressed in HEK293 cells failed to reach the plasma membrane but was instead retained intracellularly (Fig. S5). The very large amounts of intracellular protein precluded detection of bestrophin-1 at the surface of HEK293 cells, and therefore it was not possible to determine whether the mutants had reduced cell surface localisation using immunofluorescence microscopy. Instead, we examined the relative abundance of WT and mutant bestrophin-1 channels at the plasma membrane of HEK293 cells using cell surface biotinylation experiments. Cells expressing WT or mutant forms of bestrophin-1 were treated with a membrane-impermeable biotinylation reagent in order to selectively modify proteins on the cell surface. The biotinylated proteins were then isolated by binding to streptavidin-agarose beads, and subjected to immunoblotting with anti-bestrophin-1 antibodies. A considerable amount of WT bestrophin-1 was observed in the streptavidin pull-downs (Fig. 4A, lane 8), consistent with it being expressed on the surface of HEK293 cells and thus accessible for biotinylation. By comparison, very little biotinylated bestrophin-1 was detected in cells expressing the ARB mutants (Fig. 4A, lanes 9-12), despite comparable biotinylation efficiency, as demonstrated by recovery of biotinylated transferrin receptor (TfR) (Fig. 4A, lanes 7-12). Thus as observed in MCDKII cells by immunofluorescence microscopy (Fig. 1), much less of the ARB-associated bestrophin-1 mutant proteins were present at the plasma membrane of HEK293 cells compared with the WT bestrophin-1. These data provide evidence that the decreased Cl^−^ conductance function of mutant bestrophin-1 observed in HEK293 cells results from lack of cell surface expression of the mutant proteins. We next examined whether 4PBA was able to increase levels of mutant bestrophin-1 at the plasma membrane of HEK293 cells, as seen in MDCKII cells. Treatment of cells expressing p.M325T bestrophin-1 with 4PBA apparently increased the amount of bestrophin-1 that could be biotinylated by ∼twofold without obviously affecting the WT protein (Fig. 4B). Biotinylation of the other three ARB mutants was also increased following 4PBA treatment (data not shown). However, the efficiency of bestrophin-1 biotinylation was low, most likely owing to the small size of its extracellular domains; only 14 out of 585 amino acids are predicted to be exposed on the cell surface, (Kane Dickson et al., 2014) and only one of these has a free primary amine group (lysine 262) available for biotinylation. Thus, this technique might not be sufficiently sensitive to accurately quantify differences in cell surface expression of bestrophin-1 following 4PBA treatment.

In order to determine whether the mutant bestrophin-1 treated with 4PBA was functional, we cultured HEK293 cells expressing p.M325T bestrophin-1 in the presence of 4PBA for 24 h prior to measuring Cl^−^ conductance by whole-cell patch-clamp analysis. Remarkably, treatment with 2.5 mM 4PBA for 24 h restored WT-like whole-cell current responses in cells expressing the p.M325T mutant protein (Fig. 3E,F). Cl^−^ conductance was significantly higher in 4PBA-treated cells compared with untreated cells at all holding potentials (Fig. 3F) and at +80 mV, at which the Cl^−^ currents are greatest, conductance in cells expressing p.M325T was increased from 48.9±23.1 mA in the absence of 4PBA to 1118.7±330.8 pA following 4PBA treatment (Fig. 3E,F). In contrast, 4PBA treatment did not significantly affect Cl^−^ currents in cells expressing WT bestrophin-1 (compare Fig. 3A and B; Fig. 3C). Although the Cl^−^ conductance induced by p.M325T in the presence of 4PBA (1118.7±330.8 pA) seems to be higher than that induced by WT bestrophin-1 (580.6±98.7 pA), the difference was not statistically significant (*P*=0.469). We also examined the effect of 4PBA on the three other ARB-associated mutant proteins and found that restoration of channel activity could also be achieved for p.L41P, p.R141H and p.R202W bestrophin-1 (Fig. 3I; Fig. S4). Hence, at a holding potential of +80 mV, Cl^−^ conductance in cells expressing these mutant proteins was significantly increased in cells treated with 2.5 mM 4PBA compared with untreated cells (Fig. 3I). Although Cl^−^ conductance by the mutant channels was increased by treatment of cells with 4PBA, the mean current-voltage relationships show that the Cl^−^ reversal potentials were unaltered by the compound (Fig. 3C,F; Fig. S4). These findings are important as they show that the mutant bestrophin-1 channels rescued by 4PBA are functional and have similar Cl^−^ conducting properties to the WT protein.

**Fig. 5.**
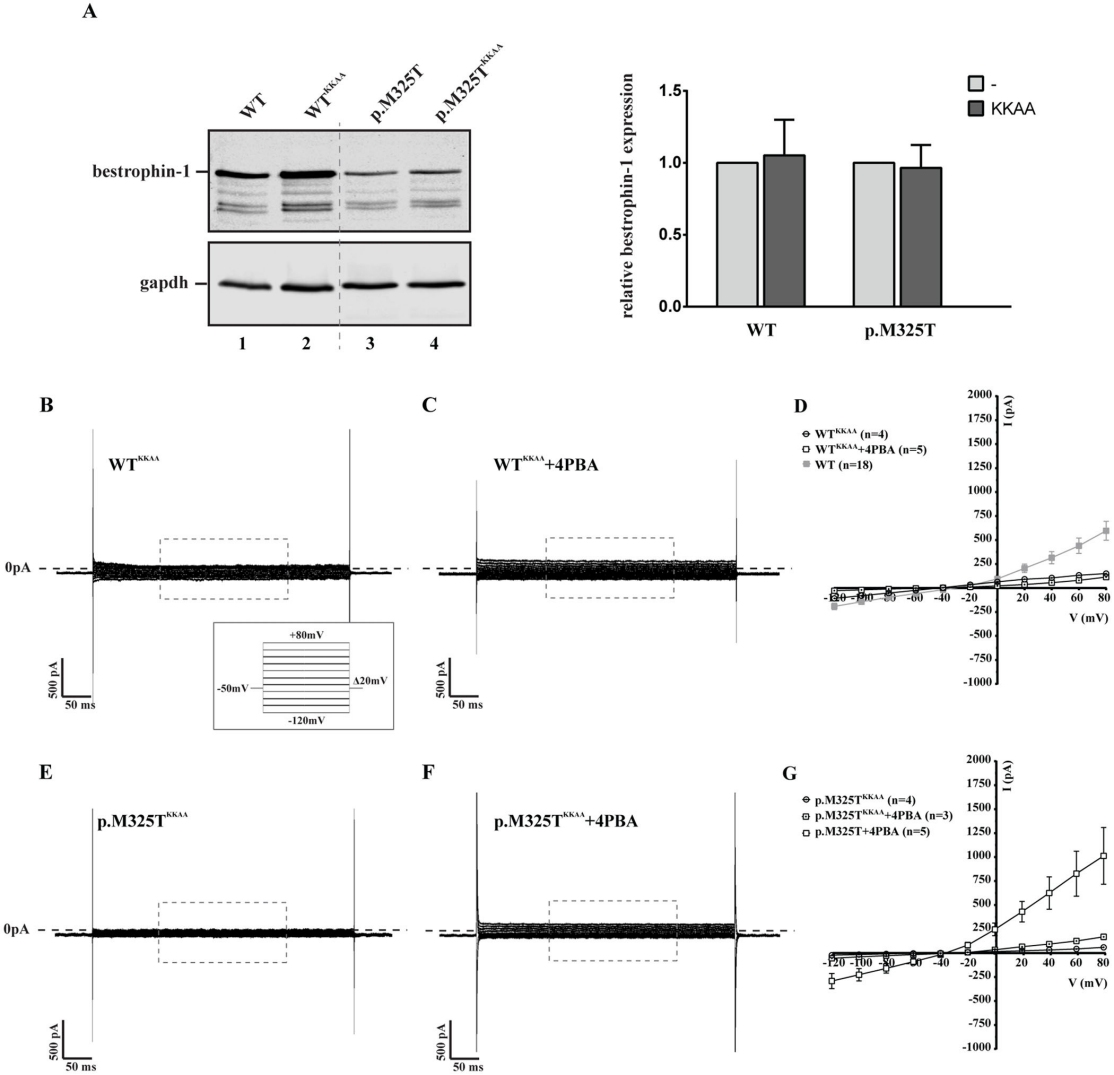
**Effect of 4PBA on the expression and activity of ER retained WT and p.M325T bestrophin-1.** (A) Western blot analysis and quantification of whole-cell lysates of HEK293 cells transiently transfected with WT, WT^KKAA^, p.M325T or p.M325T^KKAA^ bestrophin-1 constructs showed that the addition of a retention signal did not alter the steady-state levels of bestrophin-1 protein. Data are presented relative to untagged WT and p.M325T bestrophin-1, which have been normalised to 1. (B-G) Representative whole-cell current responses of HEK293 cells transiently transfected with WT^KKAA^ or p.M325T^KKAA^ bestrophin-1 constructs before (B,E) and after (C,F) 4PBA treatment. Recordings were taken from −120 mV to +80 mV in Δ20 mV steps of 450 ms each (voltage protocol shown in B). The holding potential was −50 mV. The mean current/voltage relationship (I/V) is shown for cells transfected with WT^KKAA^ or p.M325T^KKAA^ with and without 4PBA treatment (D,G). Also plotted are the I/V relationships for control untagged WT and p.M325T proteins treated with 4PBA. Results are presented as mean±s.e.m.

Whole-cell patch clamp only records currents that flow through channels in the cell membrane (Gandini et al., 2014). As 4PBA is known to function as a chemical chaperone, the simplest interpretation of these findings is that 4PBA promotes folding of each bestrophin-1 mutant protein to a native-like conformation that is no longer retained by intracellular quality control systems, leading to increased expression of functional channels at the cell surface. However, whilst 4PBA treatment increased the Cl^−^ current induced by the mutant proteins to values similar to those observed for the WT protein (Fig. 3I), the amount of mutant bestrophin-1 detected at the surface was lower than that of the WT (Fig. 4B). This apparent lack of correlation between levels of bestrophin-1 at the cell surface and Cl^−^ conductance is most likely the result of inherent differences in the techniques used to measure these parameters. Hence, data obtained by analysis of the whole cell population (biotinylation) might not be directly comparable with analysis of individual highly expressing cells (patch clamp). However, we also considered the possibility that the increased Cl^−^ conductance in 4PBA treated cells could result from an effect of 4PBA on intracellular bestrophin-1 rather than increased transport to the cell surface. To this end, we placed a dilysine ER-retrieval motif at the C-terminus of bestrophin-1 in order to prevent transport to the cell surface, allowing us to test whether restoration of Cl^−^ conductance function was indeed the result of improved trafficking of mutant bestrophin-1. Despite being expressed at comparable levels with the WT protein (Fig. 5A), Cl^−^ currents in cells expressing WT^KKAA^ bestrophin-1 were greatly reduced compared with those induced by the WT protein (Fig. 5B,D). These results are significant as they demonstrate that Cl^−^ conductance is specifically induced by cell surface localised bestrophin-1. Importantly, addition of the ER retrieval motif abolished the effect of 4PBA on mutant bestrophin-1, as treatment with 4PBA failed to restore Cl^−^ conductance in cells expressing p.M325T^KKAA^ bestrophin-1 (Fig. 5E-G). Thus, the ability of 4PBA to rescue bestrophin-1 Cl^−^ channel activity is dependent upon the protein being transported along the secretory pathway, further supporting our hypothesis that this chemical chaperone promotes native folding and restores functional bestrophin-1 at the plasma membrane. Alternatively, 4PBA treatment might enhance the activity of mutant, but not WT, bestrophin-1 (Fig. 3A-C) independently of trafficking/localisation. In either case, our data clearly demonstrate functional rescue of mutant bestrophin-1 by treatment of cells with 4PBA. This rescue of Cl^−^ conductance carried by the ARB mutant proteins together with the maintenance of the WT reversal potential provide compelling evidence that targeting protein folding pathways with compounds such as 4PBA might offer a new therapeutic opportunity for the treatment of ARB and the other bestrophinopathies resulting from missense mutations in *BEST1*.

## DISCUSSION

In this study we have restored the function of four bestrophin-1 mutant proteins associated with ARB to that of WT protein using the chemical chaperone 4PBA. This is a significant advance and paves the way for translational research using a drug that is approved for human use.

We previously showed that nine ARB-associated bestrophin-1 mutant proteins have significantly reduced Cl^−^ conductance compared with the WT protein, and provided evidence that the loss of function was associated with protein misfolding in the secretory pathway. Only two of nine mutant proteins correctly localized to the plasma membrane and six of the nine mutant proteins were degraded by the proteasome (Burgess et al., 2008; Davidson et al., 2009, 2011). Here, we have extended this research using stably transfected MDCKII cells, a polarized epithelial cell model that represents a model of the RPE. However, as with all model systems, MDCKII cells have some limitations that might be relevant to this study, including a lack of endogenous bestrophin-1. This might also be seen as advantageous for allowing unambiguous quantification of different cell treatments on the mutant bestrophin-1 protein under study and avoiding any misinterpretation from endogenous WT protein. It has also been noted that MDCKII and RPE cells have variable sorting of MCT1 (Castorino et al., 2011). Although this will be an important consideration when choosing a basolateral membrane marker for studies using RPE cells, this fact has no adverse bearing in this study. We show that both WT and mutant ARB-associated bestrophin-1 proteins are subject to a degree of proteasomal degradation and show that the loss of function of the mutant proteins results from misfolding rather than having active site mutations. Hence, we were able to rescue the expression, localization and function of the mutant proteins by treatment of cells with 4PBA. Induction of exogenous Cl^−^ conductance in transfected cells is commonly used as a measurement of bestrophin-1 function, and we demonstrate that this activity is mediated specifically by bestrophin-1 localised at the plasma membrane by whole-cell patch clamp. Hence, we propose that 4PBA promotes native folding and forward trafficking of mutant bestrophin-1, leading to increased levels of functional protein at the cell surface. However, the ability of 4PBA to restore Cl^−^ conductance in p.M325T bestrophin-1-expressing cells was much greater than its apparent effect on the amount of bestrophin-1 at the cell surface, as detected by biotinylation. Therefore we cannot exclude the possibility that 4PBA improves the function of mutant bestrophin-1 already present at the plasma membrane, rather than promoting forward trafficking of newly synthesised protein. The mechanism through which 4PBA, a hydrophobic short chain fatty acid, functions might be related to its activity as a chemical chaperone, its ability to inhibit histone deacetylases (HDACs) leading to changes in transcription of genes involved in protein folding and/or quality control, or a combination of both. In this respect, it is noteworthy that whilst treatment with an HDAC inhibitor (tubastatin) had no discernible effect, a second chemical chaperone, TMAO (50 mM), was able to restore some Cl^−^ conductance in cells expressing mutant p.M325T bestrophin-1, albeit to a much lesser extent than 4PBA (data not shown). None of the other small molecules tested (25 nM BTZ, 1 mM TUDCA, 50 nM 17-AAG, 50 nM celastrol) significantly restored the Cl^−^ conductance of p.M325T bestrophin-1 (data not shown).

Restoration of mutant protein function using chemical chaperones has been demonstrated for several proteins including myocilin and RPE65. In a cell model of RP, chemical chaperones and proteostasis regulators reduced aggregation of the common rhodopsin p.P23H mutant, whilst pharmacological chaperones enhanced folding and reduced the dominant-negative effect of the mutant protein (Mendes and Cheetham, 2008). Chemical chaperones (glycerol and 4PBA) have been shown to enhance the low temperature rescue of disease-associated mutant RPE65 proteins expressed in ARPE-19 cells (Li et al., 2014). Misfolded mutant α1-antitrypsin fails to be excreted to the bloodstream and is retained in the ER of liver cells causing liver injury and emphysema. The addition of 4PBA to human skin fibroblasts expressing mutant α1-antitrypsin resulted in a fivefold increase in its secretion compared with untreated cells. In addition, oral administration of 4PBA to PiZ mice that are transgenic for mutant human α1-antitrypsin resulted in blood levels of α1-antitrypsin increasing to 20-50% of those seen in humans and transgenic PiM mice expressing WT α1-antitrypsin (Burrows et al., 2000). There has also been promising progress in cystic fibrosis (CF) treatment using 4PBA. Treatment of primary cells from individuals with CF restored forskolin-activated Cl^−^ secretion and the post-translational modification of ΔF508-CFTR (Rubenstein et al., 1997), and *in vivo*, orally administered 4PBA improved the nasal potential difference response in CF patients with minimal side-effects (Zeitlin et al., 2002). In a transgenic mouse model of epilepsy expressing a secretion-defective mutant form of human LGI1, intraperitoneal injection of 4PBA restored the binding of mutant LGI1 protein to its receptor ADAM22 and prevented the increased seizure susceptibility of the mouse model (Yokoi et al., 2015). 4PBA has also been tested for the treatment of haemoglobin diseases and a variety of solid tumours (Zeitlin et al., 2002). Thus, the possibility of using 4PBA for the long-term correction of protein folding and function for a variety of diseases seems a realistic and achievable goal, especially as the drug is approved for use in infants and adults with urea cycle disorders who need to take it daily for life (Zeitlin et al., 2002; Batshaw et al., 2001).

The crystal structures of two bestrophin-1 orthologues have been reported and reveal that bestrophin-1 is a homopentamer (Yang et al., 2014; Kane Dickson et al., 2014). Based on the structure of the chicken bestrophin-1 orthologue, the four mutations studied here lie in different segments of the bestrophin-1 protein. Leucine 41 is in an α-helix in segment S1c, and is the only residue that lies within the membrane. Arginine 141 is located towards the end of an α-helix in segment S2d and is intracellular. Arginine 202 and methionine 325 are both located in structured loops between α-helices in the intracellular part of the protein (Kane Dickson et al., 2014). None of the four mutated amino acids studied here are located close to the channel pore or to the Ca^2+^-binding residues, supporting the notion that these mutations alter protein folding rather than disrupting the active sites of the channel.

Analysis of the amino acid substitutions in the four ARB mutants studied here support our conclusion that they are likely to disrupt protein folding. Of the four residues studied, L41 is the least conserved, being, for example, a phenylalanine in rat, serine in chicken and *Xenopus*, and valine in zebrafish. A mutation matrix for transmembrane proteins gives a score of –1 for a leucine-to-proline change, indicating that this change is not often seen and implies there will be a detrimental functional consequence (Betts and Russell, 2007). Leucine is an acyclic and hydrophobic amino acid and its replacement with proline, a small ringed amino acid that uniquely connects twice to the protein backbone, might be expected to cause a kink in the S1c segment α-helix. The other three amino acid substitutions have higher mutation matrix scores, indicating the changes are seen more frequently and are functionally less damaging. Arginine 141 is conserved from human to zebrafish and the substitution of a positive polar residue with another of similar properties (histidine) is a relatively common substitution, with a score of 5 on the mutation matrix. Arginine 202 is also relatively well-conserved (it is an asparagine in fugu and zebrafish) and it might be thought that its replacement with tryptophan, which is unique in terms of its chemistry and size, would be rare (Betts and Russell, 2007). However, an arginine-to-tryptophan substitution is also relatively common, with a mutation matrix score of 5. Lastly, methionine 325 is conserved down to zebrafish and its substitution with a threonine (a non-polar hydrophobic amino acid replaced by a polar hydrophobic residue) has a score of zero on the mutation matrix. Importantly, the detrimental effects of these diverse mutations on bestrophin-1 localisation and function could all be overcome by treatment with 4PBA, suggesting that this strategy might be applicable to other disease-causing bestrophin-1 mutants.

However, despite the analysis of the amino acid substitutions, their position in the three dimensional protein structure, and our data demonstrating that the mutant proteins are misfolded and undergo proteasomal degradation, it is still difficult to formulate a unifying pathogenic mechanism for ARB. Several ARB-associated mutations are either asymptomatic in the heterozygous state (parents of individuals with ARB) or are pathogenic (individuals with Best disease). An example illustrating the ARB conundrum is the p.R141H mutation, the most common recurring mutation associated with ARB. It can be asymptomatic in some heterozygous carriers (Burgess et al., 2008; Iannaccone et al., 2011; Kinnick et al., 2011; Wittström et al., 2011), whereas in others it can associated with mildly abnormal electroretinogram (ERG) and electrooculogram (EOG) results, indicating abnormal functioning of the photoreceptors and RPE, respectively (Wittström et al., 2011). Individuals with ARB who are homozygous for p.R141H have either abnormal or severely abnormal ERGs (Boon et al., 2013). In other individuals p.R141H acts dominantly and causes Best disease (Krämer et al., 2000; Lotery et al., 2000). There are also a number of cases involving compound heterozygous mutations in conjunction with p.R141H. The second mutation can either seem benign or be associated with another bestrophinopathy. An individual with ARB has been reported with p.R141H and p.D312N (Boon et al., 2013); as discussed above for p.R141H, p.D312N can either be asymptomatic in carriers or act dominantly in individuals with Best disease (Burgess et al., 2008; Sodi et al., 2011), giving the situation where two autosomal dominant mutations occur as compound heterozygous mutations to cause an autosomal recessive disease. Similar confounding combinations with p.R141H have also been reported with p.L41P, p.Y29X, p.P233A and p.I266fsX18 (Burgess et al., 2008; Schatz et al., 2006; Wittström et al., 2011; Johnson et al., 2015). Thus, it would seem that the pathogenic mechanism not only varies from mutation to mutation, but also on what other mutation is present and as-yet-unidentified factors such as promoter strength, modifying genes and environment. Another possibility is that it is not the adult function of bestrophin-1 that is important, but what role it might have had in development (Boon et al., 2013).

The correction of expression, localization and function for four mutant bestrophin-1 proteins associated with an inherited eye disease using a drug that is already approved for human use is a notable milestone towards future translational research. As 4PBA has proved safe during long-term daily use, often at high doses, for children and adults alike, trials to reposition the drug to treat diseases resulting from protein misfolding might be feasible within a relatively short timeframe. This research will be an exemplar for the use of small molecules to rescue other misfolded proteins of the RPE and retina that cause retinal degenerations.

## MATERIALS AND METHODS

### Cell culture

MDCKII cell lines were cultured at 37°C, 8% CO_2_ in Dulbecco's modified Eagle's Medium (DMEM) (Gibco, Paisley, UK) supplemented with 2 mM L-Glutamine (Sigma, Gillingham, UK), 0.1 mM MEM non-essential amino acids (Sigma) and 10% (v/v) heat-inactivated foetal bovine serum (Sigma). HEK293 cells were cultured at 37°C, 5% CO_2_ in DMEM without sodium pyruvate (Sigma) supplemented with 10% (v/v) heat-inactivated foetal bovine serum.

T-REx Flp-In MDCK II cells (a kind gift from Dr Joris Robben, Radboud University, Nijmegen, The Netherlands) and HEK293 cells (purchased from ATCC) were tested every three months to ensure they were free from mycoplasma using the Minerva Biolabs Venor GeM kit (Cambio, Cambridge, UK).

### Transient transfection

HEK293 cells were co-transfected with a bestrophin-1 construct and GFP at a final concentration of 0.45 µg ml^−1^ and 0.15 µg ml^−1^, respectively using Fugene HD transfection reagent (Promega, Southampton, UK) in OptiMEM (Gibco). Cells were incubated for 6-8 h at 37°C before replacing transfection medium with complete DMEM.

### Cloning and generation of stable cell lines

The ARB-associated mutations p.L41P, p.R141H, p.R202W, and p.M325T were cloned into the expression vector pcDNA5/FRT/TO (Life Technologies, Paisley, UK). These plasmids were co-transfected with pOG44 into T-REx Flp-In MDCK II cells using Lipofectamine LTX with Plus Reagent transfection reagent (Life Technologies). Stably expressing colonies were selected in the presence of 200 µg ml^−1^ hygromycin.

### RT-PCR

Total RNA was extracted using TRIzol Reagent (Fisher Scientific, Loughborough, UK) according to manufacturer's instructions. The precipitated RNA was washed to remove impurities, dried, resuspended in DNase/RNase-free water and DNase treated with TURBO DNA-free kit (Fisher Scientific). First-strand cDNA was generated form 2 μg of RNA by using the High-Capacity RNA-to-cDNA kit (Fisher Scientific) according to manufacturer's instructions. The standard PCR reaction included 1× ReddyMix custom PCR Master Mix (Abgene, Fisher Scientific), 0.5 μM forward and reverse primers, 20 ng of DNA and Milli-Q water (Millipore, Livingston, UK). The cycling parameters were an initial denature at 94°C for 5 min followed by 15 cycles of 94°C for 30 s, 60°C for 30 s and 72°C for 60 s. The reaction was completed with a cycle of 72°C for 10 min.

### Treatment with inhibitors and/or small molecules

Cells were incubated with 10 μM proteasomal inhibitor II (PSII, (benzyloxycarbonyl)-Leu-Leu-phenylalaninal) (Merck Millipore, Nottingham, UK) or a combination of 100 μM leupeptin (Enzo Life Sciences, Exeter, UK) and 1 µg ml^−1^ of pepstatin A (Sigma) for 5-6 h. When 25 nM Bortezomib (Velcade) (Selleck Chemicals, Stratech, Newmarket, UK) was used the incubation time was 16-24 h. Small molecules [2.5 mM 4PBA (Sigma), 50 mM TMAO (Sigma), 1 mM TUDCA (Calbiochem, Merck Millipore), 50 nM 17-AAG (Sigma), 50 nM celastrol (Sigma)] were added to the cultured media 24 h prior to harvesting, fixation or patch clamp analysis.

### Whole-cell patch clamp

Whole cell voltage-clamp recordings were performed using borosilicate glass capillaries GC100F-10 (Harvard Apparatus, Edenbridge, UK) that were fire-polished to a resistance of 1.3-2 MΩ. The electrophysiology setup consisted of a MultiClamp 700B amplifier controlled by pCLAMP 10.4 (Molecular Devices, Wokingham, UK). Recordings were sampled at 20 kHz and filtered online at 10 kHz. The intracellular solution (317.6 osmol) contained: 20 mM CsCl, 10 mM EGTA, 7.2 mM CaCl_2_, 2 mM MgCl_2_, 10 mM HEPES, 10 mM glucose, 110 mM Cs aspartate, adjusted to pH 7.2 with CsOH. The extracellular solution (339 osmol) contained: 140 mM NaCl, 2 mM CaCl_2_, 1 mM MgCl_2_, 10 mM HEPES, 10 mM glucose, 30 mM mannitol, adjusted to pH 7.4 with NaOH. Only cells showing a GFP signal were used for assessment of current magnitudes.

### Biotinylation assay

HEK293 cells were co-transfected with either WT or mutant bestrophin-1 constructs and GFP. After treatment with the compound of interest, cells were pre-chilled on ice and labelled with 0.5 mg ml^−1^ EZ-Link Sulfo-NHS-SS-biotin (Pierce Biotechnology, Thermo Fisher Scientific, Cramlington, UK) in PBS/CM (10 mM Na_2_HPO_4_, 2 mM KH_2_PO_4_, 137 mM NaCl, 2.7 mM KCl, 1 mM CaCl_2_, 1 mM MgCl_2_ pH 7.4) for 30 min. Cells were then washed with PBS/CM and 50 mM NH_4_Cl in PBS/CM was used to quench unreacted biotin. Cells were lysed in biotin lysis buffer (50 mM Tris pH 7.4, 150 mM NaCl, 5 mM EDTA, 1.25% Triton X-100, 0.25% SDS, 1 mM PMSF) and lysates were centrifuged at 8000×***g*** for 15 min to remove non-soluble debris. 10% of the supernatant was kept for control of total protein loading and the rest of the lysate was incubated with NeutrAvidin beads (Thermo Fisher Scientific) for 2.5 h. Beads were then washed three times in biotin lysis buffer, resuspended in 2× SDS sample buffer and analysed by SDS-PAGE and western blotting.

### SDS-PAGE and western blotting

Protein samples were extracted either in SDS sample buffer with 100 mM DTT to reduce disulphide linkages or in biotin lysis buffer. Samples were heated for 10 min at 70°C, loaded onto an 8-10% Tris-glycine polyacrylamide gel and run alongside ColorPlus pre-stained protein marker (broad range 7-175 kDa, New England Biolabs, Hitchin, UK). SDS-PAGE gels were transferred onto nitrocellulose membranes (LI-COR Biosciences, Cambridge, UK) by wet transfer at 300 mA for 1 h. Membranes were blocked in 5% milk powder (w/v) in TBS for 1 h before incubation with primary antibodies anti-bestrophin-1 (clone E6-6) (1:4000; NB300-164, Novus Biological, Cambridge, UK) and rabbit polyclonal to beta tubulin (1:4000; ab6046, Abcam, Cambridge, UK) in 2% milk-TBS+0.01% NaN_3_ solution overnight at 4°C, with constant mixing. Membranes were washed three times in TBS before labelling with fluorescently labelled secondary antibodies IRDye 800CW donkey anti-mouse (1:10,000; 925-32212) and IRDye 680CW donkey anti-rabbit (1:10,000; 925-68071) (both from LI-COR) in 2% milk-TBS for 1 h at room temperature (RT); then scanned using an Odyssey Infrared Imaging System (LI-COR) and quantified using Odyssey Sa software (LI-COR). Quantification of immunoblots was performed by using the Image Studio Ver.5.0 software (LI-COR).

### Confocal microscopy

MDCKII grown on 0.4 μm Transwell polyester membrane inserts (Corning, Sigma) were washed with PBS, fixed with 3% paraformaldehyde (w/v in PBS) for 20 min at RT and permeabilised with 0.1% Triton X-100 for 10 min. Primary antibodies were incubated for 1 h at room temperature in PBS at the following dilutions: mouse monoclonal anti-bestrophin-1 (E6-6) at 1:500 (NB300-164, Novus Biological, Cambridge, UK) and rabbit polyclonal to monocarboxylic acid transporter1 (MCT1) at 1:500 (ab85021, Abcam). Secondary antibodies Alexa Fluor 488 donkey anti-mouse (1:500; A-21202) and Alexa Fluor 594 donkey anti-rabbit (1:500; A-21207) (both from Life Technologies) were incubated for 30 min at room temperature with 100 ng ml^−1^ 4,6-diamidino-2-phenylindoledihydrochloride (DAPI). Transwell filters were cut out from the inserts and mounted onto microscope slides with 7 µl of mowiol solution or Prolong Gold anti-fade reagent (Life Technologies) and sealed with nail varnish. Images were taken using a Nikon C1 confocal on an upright 90i microscope with a 60×/1.40 Plan Apo objective and 3× confocal zoom. The confocal settings were as follows: pinhole 30 μm, scan speed 400 Hz unidirectional, format 1024×1024. Images for DAPI, FITC and Texas Red were excited with the 405 nm, 488 nm and 543 nm laser wavelengths, respectively. When acquiring 3D optical stacks the confocal software was used to determine the optimal number of *Z*-sections. Only the maximum intensity projections of these 3D stacks are shown in the results.

### Data analysis

Quantitative data collected from at least three separate experiments were plotted as means with error bars indicating standard error of the mean (±s.e.m.). Statistically significant differences among groups were identified by one-way or two-way ANOVA, followed respectively by Bonferroni or Turkey multi-comparison test, using GraphPad Prism 2D graphing and statistics software (GraphPad, La Jolla, USA).
